# 3-Benzyl-6-benzyl­amino-1-methyl-5-nitro-1,2,3,4-tetra­hydro­pyrimidine

**DOI:** 10.1107/S160053681000348X

**Published:** 2010-02-03

**Authors:** M. Kannan, P. Manivel, M. Sarathbabu, R. Sathishkumar, H. Surya Prakash Rao, R. Krishna

**Affiliations:** a Centre for Bioinformatics, Pondicherry University, Puducherry 605 014, India; b Department of Chemistry, Pondicherry University, Puducherry 605 014, India; c Solid State and Structural Chemistry Unit, Indian Institute of Science, Bangalore 560 012, India

## Abstract

In the title compound, C_19_H_22_N_4_O_2_, the tetra­hydro­pyrimidine ring adopts an envelope conformation (with the N atom connected to the benzyl group representing the flap). This benzyl group occupies a quasi-axial position. The two benzyl groups lie over the tetra­hydro­pyridimidine ring. The amino group is a hydrogen-bond donor to the nitro group.

## Related literature

For the biological activity of tetra­hydro­pyrimidine derivatives, see: Atwal *et al.* (1991 ▶); Jauk *et al.* (2000 ▶); Messer *et al.* (1997 ▶). For the synthesis of the title compound, see: Chanda *et al.* (2004 ▶). For conformational anlysis, see: Cremer & Pople (1975 ▶).
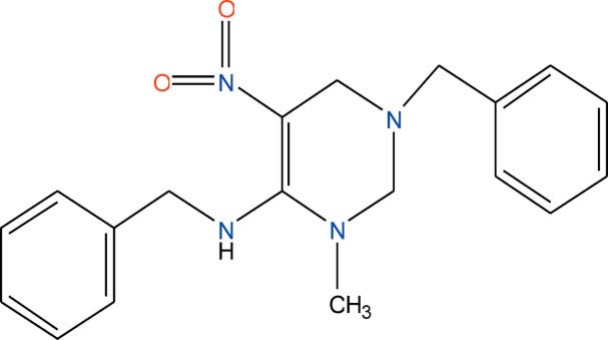


## Experimental

### 

#### Crystal data

C_19_H_22_N_4_O_2_
*M* *_r_* = 338.41
Trigonal, 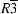

*a* = 29.2634 (12) Å
*c* = 10.4916 (8) Å
*V* = 7780.8 (7) Å^3^
*Z* = 18
Mo *K*α radiation
μ = 0.09 mm^−1^
*T* = 292 K
0.28 × 0.23 × 0.19 mm

#### Data collection

Oxford Diffraction Xcalibur diffractometer with an Eos (Nova) detector
Absorption correction: multi-scan (*CrysAlis PRO*; Oxford Diffraction, 2009 ▶) *T*_min_ = 0.979, *T*_max_ = 0.983
28183 measured reflections
3973 independent reflections
2683 reflections with *I* > 2σ(*I*)
*R*_int_ = 0.044
Standard reflections: 0

#### Refinement

*R*[*F* ^2^ > 2σ(*F* ^2^)] = 0.067
*wR*(*F* ^2^) = 0.160
*S* = 1.08
3973 reflections
227 parameters
H-atom parameters constrained
Δρ_max_ = 0.27 e Å^−3^
Δρ_min_ = −0.24 e Å^−3^

### 

Data collection: *CrysAlis PRO* (Oxford Diffraction, 2009 ▶); cell refinement: *CrysAlis PRO*; data reduction: *CrysAlis PRO*; program(s) used to solve structure: *SHELXS97* (Sheldrick, 2008 ▶); program(s) used to refine structure: *SHELXL97* (Sheldrick, 2008 ▶); molecular graphics: *ORTEP-3 for Windows* (Farrugia, 1997 ▶); software used to prepare material for publication: *PLATON* (Spek, 2009 ▶).

## Supplementary Material

Crystal structure: contains datablocks I, global. DOI: 10.1107/S160053681000348X/ng2723sup1.cif

Structure factors: contains datablocks I. DOI: 10.1107/S160053681000348X/ng2723Isup2.hkl

Additional supplementary materials:  crystallographic information; 3D view; checkCIF report

## Figures and Tables

**Table 1:** Hydrogen-bond geometry (Å, °)

*D*—H⋯*A*	*D*—H	H⋯*A*	*D*⋯*A*	*D*—H⋯*A*
N3—H3⋯O2	0.86	1.98	2.591 (2)	127

**Fig. 1.**
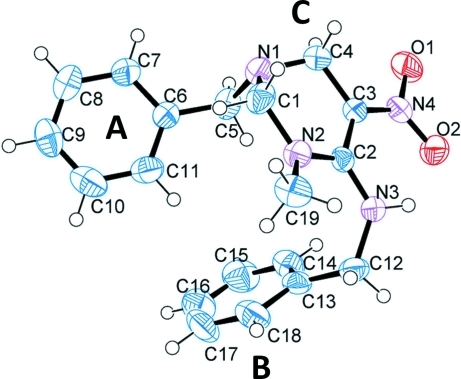
: The molecular structure of (I), showing the atom-numbering scheme and displacement ellipsoids drawn at the 50% probability level.

**Fig. 2.**
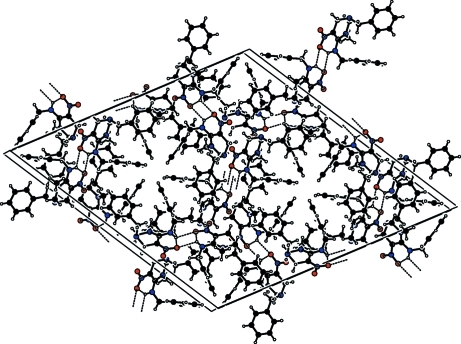
: The crystal packing of (I), showing intra and intermolecular hydrogen bonding interactions represented as dashed lines. The hydrophobic core is formed by six adjacent benzyl (B) rings.

## supplementary crystallographic information

### Comment

Dihydropyrimidines are reported to have broad range of therapeutic and pharmacological properties, such as Antihypertensive (Atwal *et al.*, 1991)and calcium channel modulators (Jauk *et al.*, 2000). Tetrahydropyrimidine derivatives found to be useful in treating cognitive and memory deficits associated with low acetylcholine levels, as found in Alzheimer disease (Messer *et al.*, 1997). Upon, considering the importance of di and tetrahydropyrimidine derivatives, we had synthesized and undertaken the single-crystal determination of the title compound.

The compound was crystallized using a solution of hexane-ethylacetate in the ratio of 7:3. The title compound, (I) was centrosymmetric. It has trigonal crystal system with hexagonal axes. It contains two planar aromatic (A & B) and one pyrimidine (C) rings (Fig. 1). The six-member (N_1_, C_1_, N_2_, C_2_,*C*_3_, C_4_) tetrahydropyrimidine ring adopts sofa conformation, with puckering parameters q2 = 0.4124 Å, q3 = -0.2504 Å, Q = 0.4825 Å, θ= 121.27° and φ =-176.41° (Cremer & Pople, 1975). Tetrahydropyrimidine ring makes dihedral angles of -176.86° with benzyl ring A and 50.26° with benzyl ring B and this contributes to the formation of sofa conformation. C_11_—H_11 _and C_g_(C_g_: centroid of C_13_, C_14_, C_15_, C_16_, C_17_) of two benzyl rings form the intermolecular C—H···π interactions with a distance of 2.862 Å. As expected, this distance was considerably lower than those observed between C—H and C of two benzyl rings which ranges from 2.884 Å to 3.449 Å. Furthermore, intermolecular hydrogen bond interactions was also observed betweeen N_3_ ···O_2_(3.056 Å), O_2_ ···O_2_ (2.882 Å), C_12_ ···O_2_ (3.184 Å) and C_19_ ···O_1_ (3.232 Å). Along with C—H ···π interaction, the intermolecular hydrogen-bonding interaction helps in stabilizing the packing of molecules in the unit cell. In addition to this, intramolecular hydrogen bonds between N_3_ and O_2_ (2.591 Å) was also observed and this interaction helps in the formation of extended three-dimensional network(Table 1). The crystal packing also shows a hydrophobic core formation between the benzyl (B) rings of each adjacent six molecules (C_16-_H_16···_H_16-_C_16_, with a distance of 2.486 Å) (Fig. 2).

### Experimental

A mixture of benzylamine (214 mg, 2.0 mmol) and paraformaldehyde (60 mg, 2.0 mmol) was stirred in methanol (2 ml) for 5 min and a solution of 1-methylamino-1-methylthio-2-nitroethylene (148 mg, 1.0 mmol) in methanol (1 mL)was added. Then, the resulting mixture was refluxed by heating at 80 *^o^*C for 2 h. After completion of the reaction (monitored byTLC), the reaction mixture was cooled in ice-water. Resulting crude solid was filtered and washed with MeOH(2 * 2 mL) to give *N*,1-Dibenzyl-3-methyl-5-nitro-1,2,3,6- tetrahydropyrimidin-4-amine(208 mg, 61%, mp = 137.5 *^o^*C) and 1,3-Dibenzyl-*N*-methyl-5-nitro-1,2,3,6- tetrahydropyrimidin-4-amine(105 mg, 31%, mp = 128.5 *^o^*C) (Chanda *et al.* 2004). X-ray worthy crystals was obtained by recrystallizing from a solution of hexane-ethyl acetate (7:3)and the X-ray data was collected at 292 K on a Oxford diffraction-Nova-1 with graphite mono chromate Mo/Kα radiation (0.71073 Å).

### Refinement

The structure was solved by direct method using *SHELXS*– 97 and refinement was done by full-matrix least-squares procedure on F2 using *SHELXL*– 97. The non-hydrogen atoms were refined anisotropically whereas hydrogen atoms were refined isotropically. The H atoms were geometrically placed (N—H = 0.86 Å, and C—H=0.93–0.97 Å) and refined as riding with *U*_iso_(H) = 1.2–1.5 *U*_eq_ (parent atom).

### Crystal data

**Table:**

C_19_H_22_N_4_O_2_	*D*_x_ = 1.300 Mg m^−^^3^
*M**_r_* = 338.41	Mo *K*α radiation, λ = 0.71073 Å
Trigonal, *R*3	Cell parameters from 28183 reflections
Hall symbol: -R 3	θ = 1.4–28.0°
*a* = 29.2634 (12) Å	µ = 0.09 mm^−^^1^
*c* = 10.4916 (8) Å	*T* = 292 K
*V* = 7780.8 (7) Å^3^	Rectangle, yellow
*Z* = 18	0.28 × 0.23 × 0.19 mm
*F*(000) = 3240	

### Data collection

**Table:**

Oxford Diffraction Xcalibur diffractometer with an Eos (Nova) detector	3973 independent reflections
Radiation source: Enhance (Mo) X-ray Source	2683 reflections with *I* > 2σ(*I*)
graphite	*R*_int_ = 0.044
ω scans	θ_max_ = 28.0°, θ_min_ = 1.4°
Absorption correction: multi-scan (*CrysAlis PRO*; Oxford Diffraction, 2009)	*h* = −38→38
*T*_min_ = 0.979, *T*_max_ = 0.983	*k* = −38→38
28183 measured reflections	*l* = −13→13

### Refinement

**Table:**

Refinement on *F*^2^	Primary atom site location: structure-invariant direct methods
Least-squares matrix: full	Secondary atom site location: difference Fourier map
*R*[*F*^2^ > 2σ(*F*^2^)] = 0.067	Hydrogen site location: inferred from neighbouring sites
*wR*(*F*^2^) = 0.160	H-atom parameters constrained
*S* = 1.08	*w* = 1/[σ^2^(*F*_o_^2^) + (0.0687*P*)^2^ + 3.8739*P*] where *P* = (*F*_o_^2^ + 2*F*_c_^2^)/3
3973 reflections	(Δ/σ)_max_ < 0.001
227 parameters	Δρ_max_ = 0.27 e Å^−^^3^
0 restraints	Δρ_min_ = −0.24 e Å^−^^3^

### Special details

**Table:**

Geometry. All e.s.d.'s (except the e.s.d. in the dihedral angle between two l.s. planes) are estimated using the full covariance matrix. The cell e.s.d.'s are taken into account individually in the estimation of e.s.d.'s in distances, angles and torsion angles; correlations between e.s.d.'s in cell parameters are only used when they are defined by crystal symmetry. An approximate (isotropic) treatment of cell e.s.d.'s is used for estimating e.s.d.'s involving l.s. planes.
Refinement. Refinement of *F*^2^ against ALL reflections. The weighted *R*-factor *wR* and goodness of fit *S* are based on *F*^2^, conventional *R*-factors *R* are based on *F*, with *F* set to zero for negative *F*^2^. The threshold expression of *F*^2^ > σ(*F*^2^) is used only for calculating *R*-factors(gt) *etc*. and is not relevant to the choice of reflections for refinement. *R*-factors based on *F*^2^ are statistically about twice as large as those based on *F*, and *R*- factors based on ALL data will be even larger.

### Fractional atomic coordinates and isotropic or equivalent isotropic displacement parameters (Å^2^)

**Table:**

	*x*	*y*	*z*	*U*_iso_*/*U*_eq_	
N3	0.39006 (6)	0.15310 (6)	0.51966 (16)	0.0398 (4)	
H3	0.3600	0.1495	0.5406	0.048*	
N2	0.42905 (6)	0.10207 (6)	0.47194 (16)	0.0402 (4)	
O2	0.31664 (6)	0.11208 (6)	0.69111 (16)	0.0545 (4)	
N4	0.33057 (6)	0.07772 (7)	0.71280 (16)	0.0417 (4)	
C2	0.39627 (7)	0.11153 (7)	0.54251 (18)	0.0328 (4)	
O1	0.30683 (6)	0.04390 (7)	0.79948 (16)	0.0617 (5)	
N1	0.43266 (7)	0.04487 (7)	0.63639 (18)	0.0456 (4)	
C4	0.37971 (8)	0.03212 (8)	0.6772 (2)	0.0493 (5)	
H4A	0.3766	0.0265	0.7687	0.059*	
H4B	0.3538	−0.0004	0.6366	0.059*	
C3	0.36820 (7)	0.07530 (7)	0.64406 (19)	0.0364 (4)	
C6	0.52871 (8)	0.09734 (8)	0.68224 (19)	0.0410 (5)	
C13	0.48157 (8)	0.22891 (7)	0.5254 (2)	0.0431 (5)	
C12	0.42799 (8)	0.20390 (8)	0.4635 (2)	0.0449 (5)	
H12A	0.4142	0.2278	0.4707	0.054*	
H12B	0.4317	0.1989	0.3735	0.054*	
C5	0.47407 (8)	0.08550 (9)	0.7173 (2)	0.0489 (5)	
H5A	0.4672	0.0739	0.8053	0.059*	
H5B	0.4724	0.1177	0.7108	0.059*	
C1	0.43797 (9)	0.05758 (9)	0.5043 (2)	0.0484 (5)	
H1A	0.4130	0.0266	0.4570	0.058*	
H1B	0.4732	0.0666	0.4770	0.058*	
C7	0.54609 (9)	0.06237 (9)	0.7111 (2)	0.0515 (5)	
H7	0.5228	0.0300	0.7481	0.062*	
C14	0.48633 (10)	0.23062 (9)	0.6569 (2)	0.0556 (6)	
H14	0.4561	0.2158	0.7070	0.067*	
C8	0.59728 (10)	0.07457 (11)	0.6862 (2)	0.0595 (6)	
H8	0.6081	0.0504	0.7059	0.071*	
C11	0.56396 (10)	0.14432 (9)	0.6243 (2)	0.0561 (6)	
H11	0.5529	0.1680	0.6012	0.067*	
C9	0.63221 (10)	0.12213 (11)	0.6324 (2)	0.0613 (7)	
H9	0.6670	0.1309	0.6180	0.074*	
C10	0.61539 (11)	0.15683 (10)	0.5999 (3)	0.0665 (7)	
H10	0.6387	0.1888	0.5615	0.080*	
C18	0.52736 (10)	0.25208 (9)	0.4537 (3)	0.0607 (7)	
H18	0.5255	0.2521	0.3652	0.073*	
C17	0.57623 (10)	0.27535 (10)	0.5138 (4)	0.0783 (9)	
H17	0.6068	0.2907	0.4650	0.094*	
C16	0.57985 (12)	0.27599 (11)	0.6432 (4)	0.0806 (9)	
H16	0.6127	0.2915	0.6824	0.097*	
C15	0.53505 (12)	0.25380 (11)	0.7148 (3)	0.0744 (8)	
H15	0.5374	0.2543	0.8032	0.089*	
C19	0.44266 (10)	0.12018 (9)	0.3407 (2)	0.0525 (6)	
H19A	0.4158	0.1260	0.3051	0.079*	
H19B	0.4452	0.0939	0.2915	0.079*	
H19C	0.4759	0.1525	0.3391	0.079*	

### Atomic displacement parameters (Å^2^)

**Table:**

	*U*^11^	*U*^22^	*U*^33^	*U*^12^	*U*^13^	*U*^23^
N3	0.0340 (8)	0.0380 (9)	0.0488 (10)	0.0191 (7)	0.0041 (7)	0.0058 (7)
N2	0.0425 (9)	0.0402 (9)	0.0393 (9)	0.0217 (8)	0.0041 (7)	−0.0024 (7)
O2	0.0521 (9)	0.0546 (9)	0.0664 (10)	0.0338 (8)	0.0166 (8)	0.0059 (8)
N4	0.0327 (8)	0.0406 (9)	0.0442 (10)	0.0125 (7)	0.0024 (7)	0.0018 (8)
C2	0.0275 (9)	0.0326 (9)	0.0357 (9)	0.0130 (8)	−0.0045 (7)	−0.0036 (8)
O1	0.0496 (9)	0.0641 (10)	0.0609 (10)	0.0205 (8)	0.0212 (8)	0.0233 (8)
N1	0.0432 (10)	0.0437 (10)	0.0553 (11)	0.0258 (8)	−0.0043 (8)	−0.0004 (8)
C4	0.0415 (11)	0.0362 (11)	0.0665 (14)	0.0166 (9)	0.0010 (10)	0.0095 (10)
C3	0.0321 (9)	0.0340 (10)	0.0404 (10)	0.0144 (8)	−0.0007 (8)	0.0014 (8)
C6	0.0437 (11)	0.0414 (11)	0.0397 (10)	0.0226 (9)	−0.0038 (9)	−0.0033 (9)
C13	0.0464 (12)	0.0277 (9)	0.0542 (12)	0.0178 (9)	0.0123 (10)	0.0026 (9)
C12	0.0513 (12)	0.0389 (11)	0.0482 (12)	0.0251 (10)	0.0113 (10)	0.0120 (9)
C5	0.0481 (12)	0.0572 (13)	0.0480 (12)	0.0313 (11)	−0.0037 (10)	−0.0041 (10)
C1	0.0517 (13)	0.0476 (12)	0.0546 (13)	0.0313 (11)	−0.0073 (10)	−0.0140 (10)
C7	0.0521 (13)	0.0479 (13)	0.0568 (13)	0.0267 (11)	0.0049 (11)	0.0094 (10)
C14	0.0532 (14)	0.0462 (13)	0.0585 (14)	0.0182 (11)	0.0079 (11)	−0.0032 (11)
C8	0.0637 (15)	0.0754 (17)	0.0582 (14)	0.0490 (14)	−0.0021 (12)	−0.0021 (12)
C11	0.0643 (15)	0.0424 (12)	0.0629 (15)	0.0277 (12)	0.0019 (12)	0.0040 (11)
C9	0.0448 (13)	0.0787 (18)	0.0564 (15)	0.0279 (13)	0.0064 (11)	−0.0091 (13)
C10	0.0614 (16)	0.0503 (14)	0.0671 (16)	0.0125 (12)	0.0150 (13)	0.0025 (12)
C18	0.0580 (15)	0.0430 (12)	0.0717 (16)	0.0181 (11)	0.0240 (13)	0.0032 (11)
C17	0.0443 (15)	0.0445 (14)	0.128 (3)	0.0089 (12)	0.0279 (16)	−0.0001 (16)
C16	0.0569 (17)	0.0500 (16)	0.121 (3)	0.0163 (13)	−0.0147 (18)	−0.0167 (17)
C15	0.0765 (19)	0.0558 (15)	0.0776 (19)	0.0232 (14)	−0.0147 (16)	−0.0131 (14)
C19	0.0618 (14)	0.0498 (13)	0.0398 (12)	0.0233 (11)	0.0123 (10)	−0.0044 (10)

### Geometric parameters (Å, °)

**Table:**

N3—C2	1.338 (2)	C5—H5A	0.9700
N3—C12	1.463 (2)	C5—H5B	0.9700
N3—H3	0.8600	C1—H1A	0.9700
N2—C2	1.346 (2)	C1—H1B	0.9700
N2—C19	1.458 (3)	C7—C8	1.380 (3)
N2—C1	1.490 (3)	C7—H7	0.9300
O2—O2	0.000 (5)	C14—C15	1.376 (4)
O2—N4	1.280 (2)	C14—H14	0.9300
N4—O1	1.265 (2)	C8—C9	1.370 (4)
N4—O2	1.280 (2)	C8—H8	0.9300
N4—C3	1.347 (3)	C11—C10	1.383 (4)
C2—C3	1.436 (3)	C11—H11	0.9300
N1—C1	1.423 (3)	C9—C10	1.374 (4)
N1—C4	1.465 (3)	C9—H9	0.9300
N1—C5	1.470 (3)	C10—H10	0.9300
C4—C3	1.502 (3)	C18—C17	1.390 (4)
C4—H4A	0.9700	C18—H18	0.9300
C4—H4B	0.9700	C17—C16	1.361 (5)
C6—C11	1.381 (3)	C17—H17	0.9300
C6—C7	1.385 (3)	C16—C15	1.362 (4)
C6—C5	1.502 (3)	C16—H16	0.9300
C13—C18	1.383 (3)	C15—H15	0.9300
C13—C14	1.386 (3)	C19—H19A	0.9600
C13—C12	1.506 (3)	C19—H19B	0.9600
C12—H12A	0.9700	C19—H19C	0.9600
C12—H12B	0.9700		
			
C2—N3—C12	128.11 (16)	C6—C5—H5B	108.9
C2—N3—H3	115.9	H5A—C5—H5B	107.7
C12—N3—H3	115.9	N1—C1—N2	113.99 (17)
C2—N2—C19	122.57 (17)	N1—C1—H1A	108.8
C2—N2—C1	120.59 (17)	N2—C1—H1A	108.8
C19—N2—C1	113.44 (16)	N1—C1—H1B	108.8
O2—O2—N4	0(10)	N2—C1—H1B	108.8
O1—N4—O2	118.41 (17)	H1A—C1—H1B	107.6
O1—N4—O2	118.41 (17)	C8—C7—C6	121.3 (2)
O2—N4—O2	0.00 (19)	C8—C7—H7	119.4
O1—N4—C3	119.16 (17)	C6—C7—H7	119.4
O2—N4—C3	122.39 (17)	C15—C14—C13	121.2 (2)
O2—N4—C3	122.39 (17)	C15—C14—H14	119.4
N3—C2—N2	121.55 (17)	C13—C14—H14	119.4
N3—C2—C3	121.09 (17)	C9—C8—C7	120.2 (2)
N2—C2—C3	117.36 (17)	C9—C8—H8	119.9
C1—N1—C4	108.36 (17)	C7—C8—H8	119.9
C1—N1—C5	114.36 (17)	C6—C11—C10	121.1 (2)
C4—N1—C5	112.11 (17)	C6—C11—H11	119.4
N1—C4—C3	111.80 (17)	C10—C11—H11	119.4
N1—C4—H4A	109.3	C8—C9—C10	119.5 (2)
C3—C4—H4A	109.3	C8—C9—H9	120.3
N1—C4—H4B	109.3	C10—C9—H9	120.3
C3—C4—H4B	109.3	C9—C10—C11	120.2 (2)
H4A—C4—H4B	107.9	C9—C10—H10	119.9
N4—C3—C2	122.62 (17)	C11—C10—H10	119.9
N4—C3—C4	116.93 (17)	C13—C18—C17	120.1 (3)
C2—C3—C4	120.45 (17)	C13—C18—H18	120.0
C11—C6—C7	117.7 (2)	C17—C18—H18	120.0
C11—C6—C5	121.1 (2)	C16—C17—C18	120.9 (3)
C7—C6—C5	121.2 (2)	C16—C17—H17	119.6
C18—C13—C14	117.9 (2)	C18—C17—H17	119.6
C18—C13—C12	121.5 (2)	C17—C16—C15	119.6 (3)
C14—C13—C12	120.56 (19)	C17—C16—H16	120.2
N3—C12—C13	113.37 (16)	C15—C16—H16	120.2
N3—C12—H12A	108.9	C16—C15—C14	120.3 (3)
C13—C12—H12A	108.9	C16—C15—H15	119.8
N3—C12—H12B	108.9	C14—C15—H15	119.8
C13—C12—H12B	108.9	N2—C19—H19A	109.5
H12A—C12—H12B	107.7	N2—C19—H19B	109.5
N1—C5—C6	113.41 (17)	H19A—C19—H19B	109.5
N1—C5—H5A	108.9	N2—C19—H19C	109.5
C6—C5—H5A	108.9	H19A—C19—H19C	109.5
N1—C5—H5B	108.9	H19B—C19—H19C	109.5
			
O2—O2—N4—O1	0.0 (2)	C1—N1—C5—C6	−59.3 (2)
O2—O2—N4—C3	0.0 (3)	C4—N1—C5—C6	176.85 (17)
C12—N3—C2—N2	−28.0 (3)	C11—C6—C5—N1	108.5 (2)
C12—N3—C2—C3	151.8 (2)	C7—C6—C5—N1	−73.9 (3)
C19—N2—C2—N3	−26.1 (3)	C4—N1—C1—N2	57.6 (2)
C1—N2—C2—N3	176.02 (17)	C5—N1—C1—N2	−68.2 (2)
C19—N2—C2—C3	154.08 (18)	C2—N2—C1—N1	−29.4 (3)
C1—N2—C2—C3	−3.7 (3)	C19—N2—C1—N1	170.85 (18)
C1—N1—C4—C3	−53.7 (2)	C11—C6—C7—C8	1.8 (3)
C5—N1—C4—C3	73.4 (2)	C5—C6—C7—C8	−176.0 (2)
O1—N4—C3—C2	178.79 (18)	C18—C13—C14—C15	−1.1 (3)
O2—N4—C3—C2	1.1 (3)	C12—C13—C14—C15	−179.4 (2)
O2—N4—C3—C2	1.1 (3)	C6—C7—C8—C9	0.3 (4)
O1—N4—C3—C4	−0.6 (3)	C7—C6—C11—C10	−2.2 (4)
O2—N4—C3—C4	−178.39 (18)	C5—C6—C11—C10	175.5 (2)
O2—N4—C3—C4	−178.39 (18)	C7—C8—C9—C10	−2.0 (4)
N3—C2—C3—N4	6.9 (3)	C8—C9—C10—C11	1.5 (4)
N2—C2—C3—N4	−173.30 (17)	C6—C11—C10—C9	0.6 (4)
N3—C2—C3—C4	−173.65 (18)	C14—C13—C18—C17	1.1 (3)
N2—C2—C3—C4	6.1 (3)	C12—C13—C18—C17	179.3 (2)
N1—C4—C3—N4	−157.21 (18)	C13—C18—C17—C16	−0.4 (4)
N1—C4—C3—C2	23.3 (3)	C18—C17—C16—C15	−0.3 (4)
C2—N3—C12—C13	−50.2 (3)	C17—C16—C15—C14	0.3 (4)
C18—C13—C12—N3	136.4 (2)	C13—C14—C15—C16	0.5 (4)
C14—C13—C12—N3	−45.4 (3)		

### Hydrogen-bond geometry (Å, °)

**Table:**

*D*—H···*A*	*D*—H	H···*A*	*D*···*A*	*D*—H···*A*
N3—H3···O2	0.86	1.98	2.591 (2)	127
N3—H3···O2^i^	0.86	2.50	3.056 (2)	123
N3—H3···N4	0.86	2.57	2.857 (2)	101
C12—H12A···O2^i^	0.97	2.54	3.184 (3)	124
C19—H19B···O1^ii^	0.96	2.40	3.232 (3)	145

Symmetry codes:  (i) −*x*+2/3, −*y*+1/3, −*z*+4/3; (ii) −*x*+*y*+2/3, −*x*+1/3, *z*−2/3.
